# Mulifunctional Dendritic Emitter: Aggregation-Induced Emission Enhanced, Thermally Activated Delayed Fluorescent Material for Solution-Processed Multilayered Organic Light-Emitting Diodes

**DOI:** 10.1038/srep41780

**Published:** 2017-01-31

**Authors:** Kenichi Matsuoka, Ken Albrecht, Kimihisa Yamamoto, Katsuhiko Fujita

**Affiliations:** 1 Institute for Materials Chemistry and Engineering, Kyushu University, 6-1 Kasuga koen, Kasuga, Fukuoka 816-8580, Japan; 2 Laboratory for Chemistry and Life Science, Tokyo Institute of Technology, 4259 Nagatsuta Midori-ku, Yokohama 226-8503, Japan

## Abstract

Thermally activated delayed fluorescence (TADF) materials emerged as promising light sources in third generation organic light-emitting diodes (OLED). Much effort has been invested for the development of small molecular TADF materials and vacuum process-based efficient TADF-OLEDs. In contrast, a limited number of solution processable high-molecular weight TADF materials toward low cost, large area, and scalable manufacturing of solution processed TADF-OLEDs have been reported so far. In this context, we report benzophenone-core carbazole dendrimers (GnB, n = generation) showing TADF and aggregation-induced emission enhancement (AIEE) properties along with alcohol resistance enabling further solution-based lamination of organic materials. The dendritic structure was found to play an important role for both TADF and AIEE activities in the neat films. By using these multifunctional dendritic emitters as non-doped emissive layers, OLED devices with fully solution processed organic multilayers were successfully fabricated and achieved maximum external quantum efficiency of 5.7%.

Solution processed organic light-emitting diode (OLED) has been one of the central research targets to realize low cost, large area, and scalable device production for display and lighting applications^1^,^2^. OLED devices generally employ multilayered structures of carrier transporting and emissive layers (EMLs) to effectively harvest electronically generated excitons. Though the multilayer of small organic molecules can be easily fabricated by successive vacuum deposition, solution-based construction of organic multilayers still has technical problems due to unfavorable dissolution of preceding bottom layers during additional layer deposition^3^,^4^. In addition, an EML is normally composed of a host material and a small amount of dopant to avoid concentration quenching of the dopant emission. This makes solution-based process challenging since the film forming process of mixture is complicated and the dissolution of both host and dopant needs to be considered at the same time. In fact, in many investigations of “solution processed” OLED, a part of organic materials (often electron transporting materials) was vacuum-deposited. In order to simplify the fabrication process and realize fully solution-processed multilayer OLED, it is desirable to develop solution processable emitters suitable for single component (non-doped) EML, which is also compatible for the top layer deposition from solutions.

In general, emissive molecules show weaker photoluminescence (PL) in neat films (aggregated states) than in solutions (molecularly dispersed states) due to concentration quenching. Therefore, non-doped EMLs with conventional emitters could not be as competitive as doped EMLs. In this context, unique materials having anti-concentration quenching properties known as aggregation-induced emission (AIE)^5^, in which nearly non-emissive molecules in solutions become highly emissive in the aggregated forms, or aggregation-induced emission enhancement (AIEE)^6^,^7^,^8^,^9^ (also called as aggregation-enhanced emission), where emission of molecules is enhanced in aggregated states, have been actively exploited. These materials have been utilized for conventional fluorescence OLEDs^10^,^11^,^12^,^13^,^14^, though only singlet excitons can be utilized for electroluminescence (EL) in fluorescence OLEDs. More recently, such properties have been found in some of thermally activated delayed fluorescence (TADF) materials^15^,^16^,^17^,^18^,^19^,^20^,^21^, where singlet and triplet excitons can be fully utilized in TADF-OLED^15^,^16^,^17^,^18^,^19^,^20^,^21^,^22^,^23^,^24^,^25^. However, to the best of our knowledge, fully solution processed systems in TADF-OLED devices combined with the aforementioned unique properties have not been established yet.

It has been reported that relatively high molecular weight host materials (Mw > ~1000) for phosphorescent emitters showed strong resistance to alcohol treatment, enabling the following deposition of electron transporting materials from alcoholic solutions on the EMLs (orthogonal solvent (OS) approach)^3^. In this context, dendritic materials^26^ could be good candidates for solution-based multilayer construction by OS approach because the molecular weight of dendrimers can be high enough by increasing their generation number and/or introduction of functional groups. Carbazole dendrimers have been investigated for OLED application as hole-transporting, phosphorescent host, and emissive materials particularly because of relatively-high triplet energy, hole transporting property, and good film forming characteristics^27^,^28^,^29^,^30^. Carbazole dendrons^16^,^30^,^31^,^32^ and related bicarbazole units^23^,^33^ have been incorporated in TADF materials with various electron accepting units. Here, we report benzophenone-core carbazole dendrimers (GnB, n = generation) as AIEE and TADF active emitters for non-doped EMLs in OLED devices with fully solution processed organic multilayer fabricated by OS approach.

## Results and Discussion

### Molecular design

**Figure 1:**
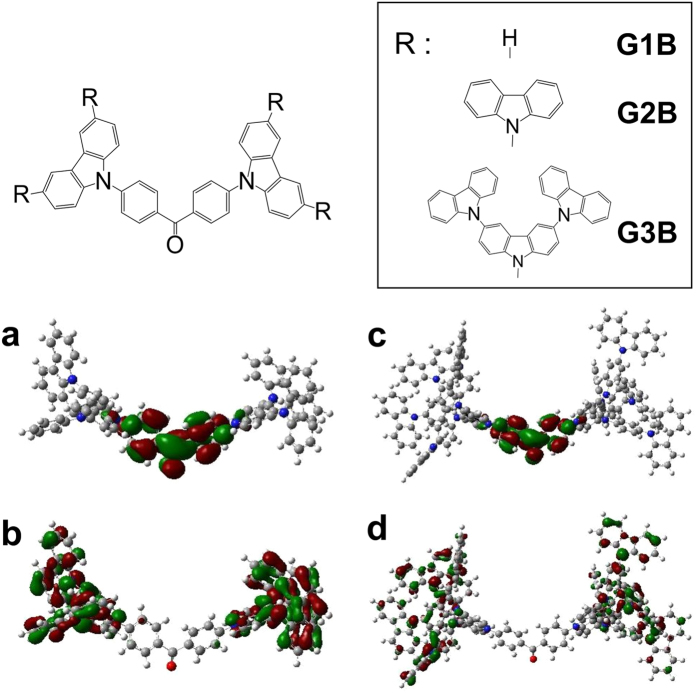
Chemical structures of GnB and calculated molecular orbitals of G2B and G3B. (**a**) G2B-LUMO, (**b**) G2B-HOMO, (**c**) G3B-LUMO, (**d**) G3B-LUMO (density functional theory at CAM-B3LYP/6-31 G (d) level).

GnB (n = 1–3) was synthesized according to the previous report^34^. Carbazole dendrimers have outer-layer electron rich potential gradient character where a highest occupied molecular orbital (HOMO) is localized at outer-layer carbazole units^34^. Appropriate choice of acceptor-type core unit places a lowest unoccupied molecular orbital (LUMO) of the carbazole dendrimer near the core to establish spatial HOMO-LUMO separation. In principle, spatial separation of HOMO and LUMO is necessary for TADF activity by reducing the energy difference between excited singlet and triplet energy level (*ΔE*_ST_)^22^. Sufficient HOMO-LUMO separation can be deduced from the calculated molecular models^35^ of G2B and G3B (Fig. 1(a–d)) showing that the HOMO and LUMO are highly localized in outer-most carbazole and benzophenone core, respectively. Optical and theoretical characterizations of G1B can be found elsewhere^23^. HOMO/LUMO levels of G2B and G3B, evaluated by photoelectron yield spectroscopy and absorption edge of the neat films, were 5.87/2.91 eV and 5.73/2.67 eV, respectively.

### Optical characteristics

**Figure 2:**
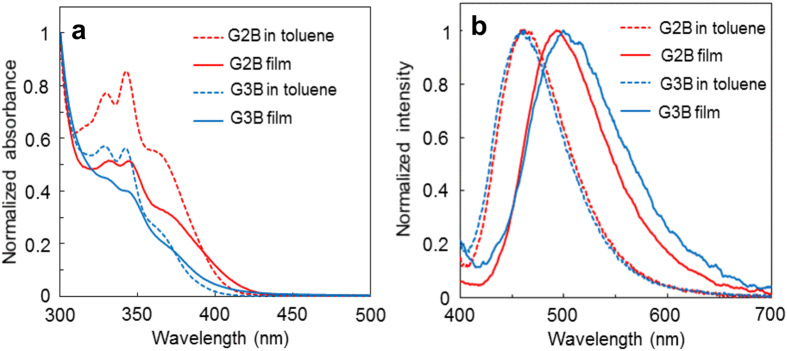
UV-vis and PL spectra of GnB solution and neat film. (**a**) Normalized UV-vis spectra and (**b**) PL spectra of GnB in toluene solution and neat film (excited at 380 nm).

GnB in toluene solution exhibited charge transfer (CT) band around 380 nm attributable to the direct HOMO-LUMO transition from carbazole units to benzophenone core (Fig. 2(a)). Absorption coefficient of the CT band decreased as the generation number n of GnB increased (Supplementary Fig. S1). The decreased CT band intensity can be explained by the increased distance between HOMO and LUMO, which makes corresponding transition difficult^30^. In PL spectra of GnB in oxygen-free toluene solution, the emission appears at around 460 nm (Fig. 2b) with PL quantum yield (PLQY) values of 23.6% (G2B) and 14.7% (G3B), respectively. Emissions from GnB are broad and strongly influenced by solvent polarity, suggesting that observed emissions are originated from CT state (Supplementary Fig. S2). Additionally, the Lippert-Mataga plot^36^,^37^ of GnB with Lippert’s solvent polarity showed linear correlation from toluene to dimethyl sulfoxide (Supplementary Fig. S3). However, in pentane, the spectra had structure, and was not on the regression line of other solvents in the Lippert-Mataga plot. This indicates that the emission of GnB in pentane is from the locally excited (LE) state, and in over polar solvents from the twisted intramolecular charge transfer (TICT) state^38^. The UV-vis spectra of GnB neat films show minor bathochromic shifts as compared to those in toluene solution by intermolecular interaction. In contrast, PL spectra of GnB films were drastically redshifted for 30–40 nm as compared to those in the toluene solution (Fig. 2(b)). This is reasonable due to the fact that PL from CT states is sensitive to external environment and intermolecular interaction. PLQY values of the GnB films under N_2_ atmosphere were 33.4% (G2B) and 21.1% (G3B). PLQY values of GnB neat films are about 1.4 times higher than those in the toluene solution.

### TADF property

*ΔE*_ST_ of GnB in toluene solution was estimated from the PLQY and transient PL decay curves at room temperature based on the reported method by Adachi’s group^39^ (Supplementary Fig. S4 and Table S1). G2B and G3B exhibit long lifetime component with *τ* = ~0.7–0.75 μs attributable to TADF, and *ΔE*_ST_ was calculated to be 0.120 eV for G2B and 0.115 eV for G3B. PLQY of the fluorescence (*Φ*_F_) and TADF components (*Φ*_TADF_) were calculated based on the relative integrated intensity of each component in the decay curves and overall PLQY (*Φ*). G2B and G3B in toluene showed small *Φ*_TADF_ of 0.5% and 1.0%, respectively. In case of the neat films, *ΔE*_ST_ was determined to be 0.12 eV for G2B and 0.10 eV for G3B based on the onset of fluorescence (300 K) and phosphorescence (77 K) spectra (Supplementary Fig. S5). The neat films showed much higher *Φ*_TADF_ of 13.4% for G2B and 13.6% for G3B as compared to those in toluene solution. Summary of the photophysical properties is shown in Table 1. *ΔE*_ST_ of G1B was reported to be 0.21 eV in a doped film^23^, i.e., when the generation of the dendrimer increases, the exchange interaction integral of the HOMO and LUMO wavefunctions decreases which leads to decrease of *ΔE*_ST_.

**Figure 3:**
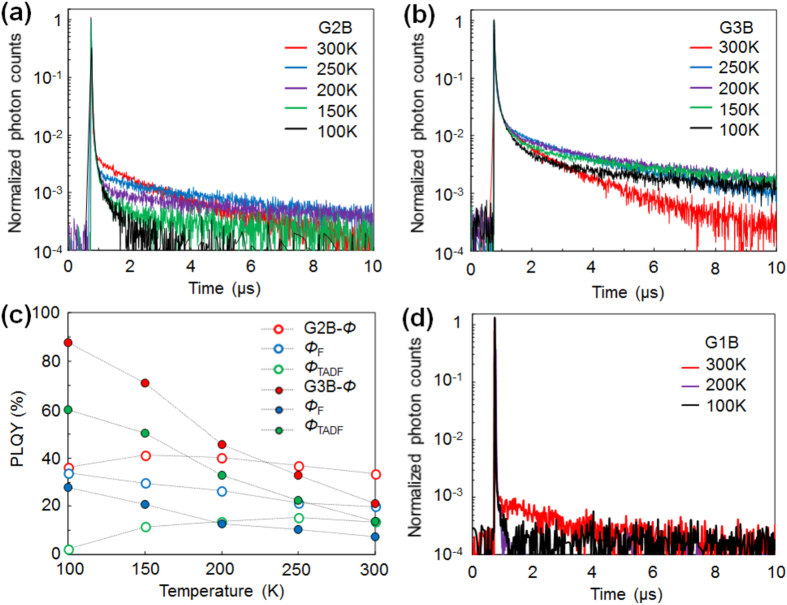
Temperature dependent PL properties of GnB neat films. Transient PL decay curves of (**a**) G2B and (**b**) G3B neat films at various temperatures. (**c**) Plot of *Φ*_F_, *Φ*_TADF_, and *Φ* of G2B and G3B neat films at various temperatures. (**d**) Transient PL decay curves of G1B neat film at various temperatures.

Figure 3(a,b) shows temperature dependence of the transient PL decay curves of GnB neat films. Both G2B and G3B films exhibit prompt component with lifetime of up to tenth of nanosecond and delayed components in sub-micro to micro second order at varied temperatures attributable to TADF contribution (Supplementary Table S2). Figure 3(c) describes the temperature dependence of *Φ*_F_, *Φ*_TADF_, and *Φ*. Delayed component of G2B showed a typical phenomenon of TADF where *Φ*_TADF_ increased as the temperature increased, while *Φ*_F_ of G2B was gradually decreased. G3B shows much stronger temperature dependence than G2B. *Φ*_F_, *Φ*_TADF_, and therefore, *Φ* gradually decreased as temperature increased (*Φ*: 87.8% at 100 K and 22.1% at 300 K). G2B show relatively smaller temperature dependence where *Φ* reached 41.1% at 150 K which is only 1.2 times higher than that at 300 K. The difference between G2B and G3B could be due to the temperature dependence of non-radiative decay process where the larger molecular size of G3B made the internal conversion of excitation energy more efficient than G2B. G1B was previously reported as a TADF emitter when doped in bis[2-(diphenylphosphino) phenyl]ether oxide matrix with a *Φ*_TADF_ of 50% at 300 K^23^. In contrast, delayed component in G1B neat film was hardly observed (Fig. 3(d)). *Φ*_TADF_ of G1B at 300 K was calculated to be only 0.004% and no delayed components were detectable at lower temperatures, indicating that the TADF activity of G1B was significantly deactivated in the aggregated state presumably due to intermolecular interaction. These results illustrate the importance of dendritic structure to exhibit TADF activity in GnB neat films.

### AIEE property

**Figure 4:**
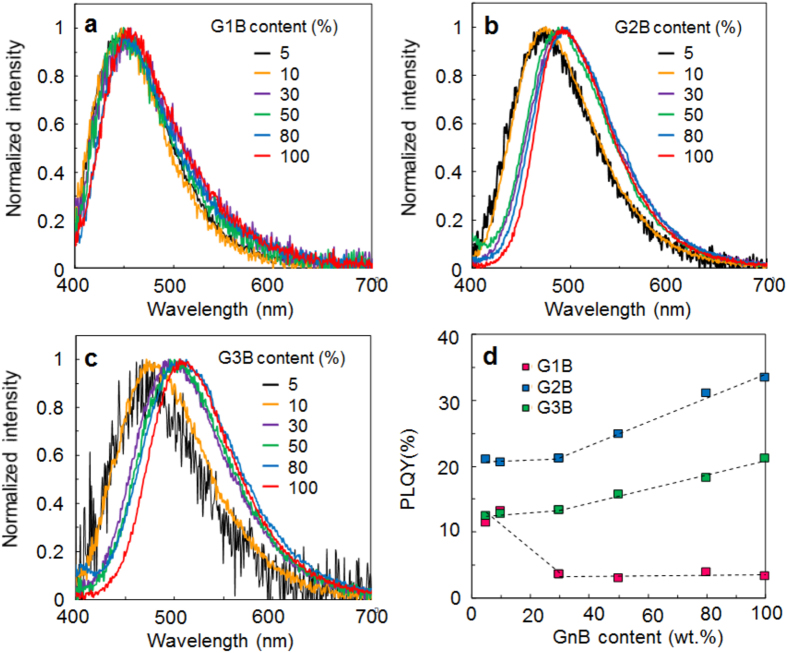
PL properties of GnB-PMMA composite films. PL spectra of (**a**) G1B-PMMA, (**b**) G2B-PMMA, (**c**) G3B-PMMA composite films (excited at 380 nm). (**d**) PLQY of GnB-PMMA composite films vs. GnB content.

The effect of intermolecular interaction on the PL property of GnB was investigated by solid state dilution experiment^40^ in poly (methyl methacrylate) (PMMA) matrix. PMMA has high triplet energy of 3.1 eV^41^ and offers rigid environment for organic molecules^42^,^43^. GnB-PMMA composite films were prepared by spincoating from tetrahydrofuran solutions containing various weight ratios of GnB and PMMA. As shown in Fig. 4(b,c), PL spectra of the composite films with low G2B and G3B contents (5, 10 wt.%) appeared relatively short wavelength region and then redshifted as the weight ratio of GnB increased due to the rise of intermolecular interaction. Besides, PLQY of G2B and G3B increased as the weight fraction increased especially above 30 wt.%. G1B was also tested as a control experiment (Fig. 4(a)). In this case, PL spectra of G1B-PMMA composite films also showed spectral redshifts as the G1B content increased, but the degree of redshift was small as compared to G2B and G3B. The PLQY value was significantly dropped at 30 wt.% content and kept low around 3% until G1B content reached 100 wt.%.

Normalized UV-vis spectra of the composite films as shown in Supplementary Fig. S7 confirmed that the spectral shape of 5 and 10 wt.% GnB films are very similar in all cases, and start to change above 30 wt.%. These results indicate that GnB molecules are well-dispersed in PMMA matrix below 10 wt.% and start to aggregate at least above 30 wt.%. The UV-vis spectral change above 30 wt.% GnB was stepwise, implying that the ratio of the aggregated GnB to the dispersed GnB was stepwisely increased. The results are in agreement with the trend of the PL properties, where G2B- and G3B-PMMA films experience large spectral redshift between 10 and 30 wt.% by the progress of aggregation and PLQY values notably increase above 30 wt.% contents. PL spectra of the composite films containing aggregated GnB with relatively low GnB content, for example 30 wt.% GnB, are broadened especially at short wavelength region as compared to that of GnB neat films. Full-width at half maximum (FWHM) of PL spectrum at 30 wt.% G2B and G3B is 103 nm and 112 nm, respectively, and these values decrease to 88 nm and 99 nm at 100 wt.% G2B and G3B. This is likely due to the contribution of dispersed GnB since dispersed GnB emits PL at short wavelength region as compared to the aggregated species. In case of G1B, PLQY is suddenly dropped at 30 wt.% and kept low up to 100 wt.% likely due to concentration quenching.

Transient PL decay curves of GnB-PMMA composite films at room temperature were analyzed to investigate the details (Supplementary Fig. S8 and Tables S3 and S4). It was found that *Φ*_TADF_ in neat films was highly enhanced by a factor of 2.7 for G2B (*Φ*_TADF_ = 4.9% at 10 wt.% and 13.4% in neat film) and 5.2 for G3B (*Φ*_TADF_ = 2.6% at 10 wt.% and 13.6% in neat film) as compared to the diluted condition. (Supplementary Fig. S8 and Table S3). In case of *Φ*_F_, relatively small enhancement (1.3 times) for G2B or reduction (0.74 times) for G3B was observed. Therefore, the observed PLQY enhancement is mainly due to the enhanced TADF emission by aggregation. Origin of AIE or AIEE activity in many studies have been considered as a result of restriction of intramolecular motions (e.g. rotations and vibrations), that create fast non-radiative decay pathway of excited state energy, by intermolecular interaction (aggregation). Actually, several TICT molecules are reported as AIE or AIEE active molecules, because the rotation (twisting motion) is suppressed by aggregation^44^,^45^,^46^,^47^. If the present case is in the similar situation, such effect would be reflected in the rate constant and quantum yield of internal conversion (*k*_IC_ and *Φ*_IC_). It was found that both *k*_IC_ and *Φ*_IC_ continuously decrease from the dispersed state to the aggregated state in both G2B (*k*_IC_ = 10.1 × 10^8^ s^−1^, *Φ*_IC_ = 61% at 10 wt.% and 3.82 × 10^7^ s^−1^, 40% in neat film) and G3B (*k*_IC_ = 5.88 × 10^7^ s^−1^, *Φ*_IC_ = 70% at 10 wt.% and 1.34 × 10^7^ s^−1^, 28% in neat film). In addition, *k*_IC_ of G2B and G3B neat films is about one order of magnitude lower than that in toluene solution (Supplementary Table S1). Concequently, reduced *k*_IC_ and *Φ*_IC_ contributed, at least in part, to improve the quantum yield of intersystem crossing (*Φ*_ISC_) and enhanced TADF emission. These observations imply that AIEE activity of G2B and G3B is due to the restricted intramolecular motions by aggregation. Above experiments clarify that the dendritic structure plays an important role for the AIEE activity of GnB. However, it is still unclear what factor differentiates the optical property between G1B and G2,3B at this stage.

We have conducted an additional experiment in small molecular host of 3,5-bis(carbazol-9-yl)benzene (mCP), whose triplet energy is 2.9 eV (Supplementary Fig. S9). In contrast to PMMA matrix, PLQY of G2B and G3B in mCP host was almost continuously increased as GnB contents decreased, reaching 40.9% and 28.5% in 10 wt.% GnB film, respectively. Transient PL decay curve analysis of 10 wt.% GnB doped mCP films shows that the *k*_IC_ values (4.02 × 10^7^ s^−1^ for G2B and 5.05 × 10^7^ s^−1^ for G3B) are lower than the values of 10 wt.% doped PMMA film, but still higher than the neat films (Supplementary Figure S10 and Table S5). The results indicate that solid aromatic media (including G2B and G3B themselves) could help to increase PLQY by restricting intramolecular motions (such as rotation) through π-π interaction. These observations exemplify that the optical property of GnB is influenced by the surrounding matrix. It should be noted that mCP is a good host material for OLED, but it is not compatible for solution-based multilayer OLED construction by OS approach used in this study^3^.

### OLED device characteristics

**Figure 5:**
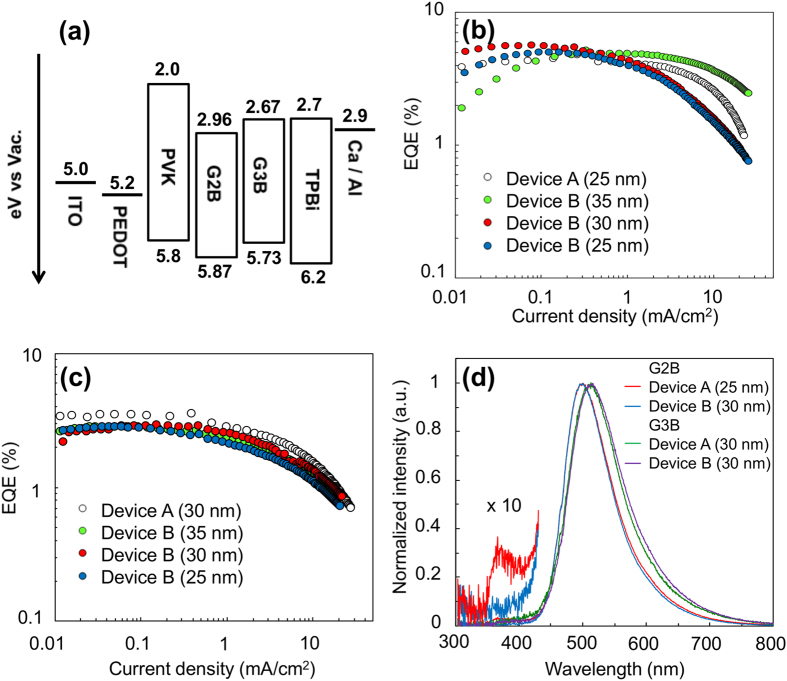
Device characteristics of OLED with GnB non-doped emitters. (**a**) Energy diagram of OLED device. EQE-current density characteristics of (**b**) G2B and (**c**) G3B OLEDs. (**d**) Normalized EL spectra of GnB OLEDs (at 10 mA/cm^2^). Parenthesis corresponds to the EML thickness.

Solution processed OLED devices with device structure of ITO/PEDOT:PSS (ca.70 nm)/PVK (ca. 20 nm)/GnB/TPBi (ca.40 nm)/Ca (10 nm)/Al (80 nm) (PEDOT:PSS = poly(3,4-ethylenedioxythiophene) polystyrene sulfonate, PVK = Poly(9-vinylcarbazole), TPBi = 1,3,5-tris(1-phenyl-1H-benzimidazol-2-yl)benzene, see Fig. 5(a) for energy diagram) were investigated. It has been reported that TPBi layer can be deposited from alcohol solutions^3^. Two types of OLED device, in which TPBi layer was fabricated either by vacuum deposition (device A) or spincoating (device B), were compared. As shown in Supplementary Fig. S10, G2B and G3B films were almost intact to alcohols, though negligible dissolution of G2B layer could be observed. In contrast, G1B film was severely dissolved. Molecular weight of G1B, G2B and G3B is 513, 1173 and 2495, respectively, and the above result is consistent with the aforementioned trend that relatively high molecular weight materials (Mw > ~1000) show good alcohol resistance^3^.

In case of device A with an optimized EML thickness of 25 nm for G2B and 30 nm for G3B, maximum external quantum efficiency (EQE_max_) of 4.8% (G2B) and 3.6% (G3B) was achieved as shown in Figs 5(b,c), S11 and Table 2. G2B-device A shows green EL centered at 500 nm with a small peak around 380 nm (Fig. 5(d)). The small peak is attributable to EL from the adjacent TPBi layer^48^, but no such peak was observed in G3B-device A. This difference originates from the incomplete hole blocking at G2B/TPBi interface due to the deeper HOMO level of G2B as compared to G3B. In case of device B, where organic layers were fully solution processed, EQE_max_ of 5.7% (G2B) and 2.9% (G3B) was achieved when EML thickness was 30 nm in both cases. No sign of EL from TPBi layer was detected in both G2B- and G3B-device B. Notably, in case of G2B, performance of device B outperformed device A in terms of EQE, current efficiency (CE), and power efficiency (PE). In addition, thick G2B film (35 nm) was found to mitigate EQE roll-off at high current density, likely due to lowered probability of deactivation processes such as triplet-triplet annihilation by reducing exciton population at constant current density^30^. The higher performance in G2B-device B could be attributed to the intermixing at G2B/TPBi interface during the TPBi deposition process where electron injection from TPBi to G2B layer was facilitated and consequently, exciton recombination at TPBi layer was suppressed.

OLED devices with doped EMLs (10 wt.% GnB in mCP, denoted as mCP-GnB) were also tested for comparison (Supplementary Figure S13). Note that electron transporting layer was vacuum-deposited for the entire examination due to solubility problem. Replacement of non-doped EML (GnB) by mCP-GnB films in the same device configuration discussed above (device A) resulted in poor device performance (EQEmax ~1.2% for mCP-G2B and ~0.8% for mCP-G3B). HOMO level of mCP (5.9 eV) is slightly higher than those of G2B and G3B, and incomplete hole blocking at mCP-GnB/TPBi interface was expected. Replacement of TPBi by 1,3-bis[3,5-di(pyridin-3-yl)phenyl]benzene (BmPyPhB) with deeper HOMO level (6.7 eV) and removal of PVK interlayer improved the device performance. The highest EQE_max_ values obtained with the devices structure of ITO/PEDOT:PSS/mCP-GnB/BmPyPhB/LiF/Al (device C) were 3.6% (mCP-G2B) and 2.2%, (mCP-G3B) respectively. These values are still lower than the non-doped EML devices. EL spectra of the devices shows emission from GnB along with a weak emission around 350 nm attributable to the fluorescence of mCP and small peaks around 460 nm possibly due to the phosphorescence of mCP^49^, indicating that a part of excitons decayed in mCP matrix and resulted in the lower EQE values. Further screening of host materials and/or entire device structures could improve the device performance of OLEDs with doped EMLs. However, such effort would be incompatible with the establishment of TADF-OLED with fully solution processed organic multilayers, which is one of the primary concerns in this study, unless the solubility problem for multilayer construction is taken into account.

In summary, a series of benzophenone-core carbazole dendrmers (GnB) was investigated toward efficient non-doped EML application in solution processed OLED. It was demonstrated that the dendritic structure contributed the multifunctionalization of benzophenone-carbazole linked materials, i.e., neat films of G2B and G3B simultaneously expressed of AIEE, TADF, and compatibility for the further deposition of the electron transporting material (TPBi) from alcohol solution, while that of G1B showed almost none of those properties. OLED devices having fully solution processed organic layers including TADF- and AIEE-active undoped EMLs were successfully fabricated and achieved EQE_max_ of 5.7%. A novel approach to endow AIEE to high molecular weight TADF materials will be beneficial for the future development of highly emissive and multifunctionalized emitters toward low-cost and efficient fully-solution processed OLED.

## Methods

### OLED fabrication and characterization

OLED devices having ITO/PEDOT:PSS (70 nm)/PVK (20 nm)/GnB/TPBI (40 nm)/Ca (10 nm)/Al (80 nm) structure were fabricated as follows: PEDOT:PSS dispersion (Clevios P VP CH8000) was spincoated on UV ozone-treated ITO-coated (150 nm thick, 10 Ω/sq.) glass substrate at 2000 rpm for 30 sec and annealed at 200 °C for 10 min, after which the substrates were brought into a globe box. PVK (0.8 wt.%, Mw 1,100,000) in *o*-dichlorobenzene was spincoated at 2000 rpm for 30 sec and annealed at 140 °C for 30 min. Toluene solutions of GnB were spincoated at 2000 rpm for 30 sec followed by 3000 rpm for 3 sec, then annealed at 100 °C for 30 min. Concentration of GnB solution was varied (5 mg/ml to 10 mg/ml) to obtain desirable film thickness. In case of device A, TPBi was vacuum deposited for 40 nm at deposition rate of 0.1–0.2 nm/s. In case of device B, alcohol solution of TPBi (6 mg/ml methanol for G2B, and 10 mg/ml ethanol for G3B) was spincoated (2000 rpm for G2B, and 1500 rpm for G3B) for 30 sec and annealed at 70 °C for 30 min. Thickness of the spincoated TPBi layer was estimated to be ca. 35–40 nm. The cathode was fabricated by vacuum deposition of Ca, followed by Al layer. The deposition rates of Ca and Al layers were 0.05 nm/s and 0.2–0.5 nm/s, respectively.

In case of device C with a device structure of ITO/PEDOT:PSS (70 nm)/mCP-GnB (30 nm)/BmPyPhB (40 nm)/LiF (0.5 nm)/Al (80 nm), PEDOT:PSS dispersion (Clevios P VP CH8000) was spincoated on UV ozone-treated ITO-coated (150 nm thick, 10 Ω/sq.) glass substrate at 2000 rpm for 30 sec and annealed at 200 °C for 10 min, after which the substrates were brought into a globe box. Toluene solutions containing mCP (6.3 mg/ml) and GnB (0.7 mg/ml) were spincoated at 2000 rpm for 30 sec, then annealed at 100 °C for 30 min. BmPyPhB was vacuum deposited for 40 nm at deposition rate of 0.1–0.2 nm/s. Finally, LiF and Al were vacuum deposited at 0.01 nm/s and 0.2–0.5 nm/s, respectively. The OLED devices were sealed with a sealing glass and an epoxy resin in a glove box. Device area was 4 mm^2^. Thickness of each organic layer was measured by AFM. Current density, voltage, and luminance characteristics of OLED devices were measured by DC voltage current source/monitor (6241 A, ADCMT) and luminance colorimeter (BM-5A,Topcon). EL spectra were recorded by photonic multichannel analyzer (PMA-11, Hamamatsu Photonics).

## Additional Information

**How to cite this article**: Matsuoka, K. *et al*. Mulifunctional Dendritic Emitter: Aggregation-Induced Emission Enhanced, Thermally Activated Delayed Fluorescent Material for Solution-Processed Multilayered Organic Light-Emitting Diodes. *Sci. Rep.* **7**, 41780; doi: 10.1038/srep41780 (2017).

**Publisher's note:** Springer Nature remains neutral with regard to jurisdictional claims in published maps and institutional affiliations.

## Supplementary Material

Supporting Information

## Figures and Tables

**Table 1:** Photophysical property of G2B and G3B in toluene solution and neat film.

Material	λ_PL_ (nm) Sol./Film	*Φ* (%) Sol./Film	*Φ*_F_ (%) Sol./Film	*Φ*_TADF_ (%) Sol./Film	ΔE_ST_ (eV) Sol./Film	S_1_/T_1_ (eV) Film
G2B	461/493	23.6/33.4	23.1/20.0	0.5/13.4	0.120/0.12	2.84/2.72
G3B	461/500	14.7/21.1	13.6/7.5	1.1/13.6	0.115/0.10	2.84/2.74

*Φ, Φ*_F_, *Φ*_TADF_ are the value measured at room temperature.

**Table 2:** Optimized device performance of device A and B.

Material	Device type	As-spun EML thickness (nm)	V_th_ at 1 cd/m^2^ (V)	EQE_max_ (λ_EL_) [% (nm)]	CE_max_ (cd/A)	PE_max_ (lm/W)	CIE (x, y)
G2B	Device ADevice B	2530	4.33.4	4.8 (500)5.7 (500)	11.314.0	7.111.5	0.25, 0.440.26, 0.48
G3B	Device ADevice B	3030	4.03.7	3.6 (513)2.9 (516)	8.77.7	6.65.7	0.28, 0.430.31, 0.50

## References

[b1] Friend R. H. . Electroluminescence in conjugated polymers. Nature 397, 121–128 (1999).

[b2] Zhong C., Duan C., Huang F., Wu H. & Cao Y. Materials and devices toward fully solution processable organic light emitting diodes. Chem. Mater. 23, 326–340 (2011).

[b3] Aizawa N. . Solution-processed multilayer small-molecule light-emitting devices with high-efficiency white-light emission. Nat. Commun. 5, 5756 (2014). 25519692 10.1038/ncomms6756

[b4] Sax S. . Efficient blue-light-emitting polymer heterostructure devices: The fabrication of multilayer structures from orthogonal solvents. Adv. Mater. 22, 2087–2091 (2010). 20544896 10.1002/adma.200903076

[b5] Mei J. . Aggregation-induced emission: Together we shine, united we soar !, Chem. Rev. 115, 11718–11940 (2015). 26492387 10.1021/acs.chemrev.5b00263

[b6] Zeng Q. . Fluorescence enhancements of benzene-cored luminophores by restricted intramolecular rotations: AIE and AIEE effects. Chem. Commun. 70–72 (2007). 10.1039/b613522f 17279264

[b7] Qin A. . Aggregation-enhanced emissions of intramolecular excimers in disturbed polyacetylenes, J. Phys. Chem. B 112, 9281–9288 (2008). 18630853 10.1021/jp800296t

[b8] Zhang X. . Piezofluorochromic properties and mechanism of an aggregation-induced emission enhancement compound containing N-Hexyl-phenothiazine and anthracene moieties. J. Phys. Chem. B 115, 7606–7611 (2011). 21591771 10.1021/jp202112e

[b9] Zhang W. . Disubstituted polyacetylenes containging photopolymerizable vinyl groups and polar ester functionality: Polymer synthesis, aggregation-enhanced emission and fluorescent pattern formation, Macromolecules 40, 3159–3166 (2007).

[b10] Chang Z. . Aggregation-enhanced emission and efficient electroluminescence of tetraphenylethene-cored luminogens. Chem. Commun. 49, 594–596 (2013). 10.1039/c2cc37928g 23212231

[b11] Yuan W. Z. . Changing the behaviour of chromophores from aggregation-caused quenching to aggregation-induced emission: Development of highly efficient light emitters in the solid state. Adv. Mater. 22, 2159–2163 (2010). 20564253 10.1002/adma.200904056

[b12] Ning Z. . Aggregation-induced emission (AIE)-active starburst triarylamine fluorophores as potential non-doped red emitters for organic light-emitting diodes and Cl_2_ gas chemodosimeter. Adv. Funct. Mater. 17, 3799–3807 (2007).

[b13] Huang J. . Similar or totally defferent: The control of conjugation degree through minor structural modifications, and deep-blue aggregation-induced emission luminogens for non-doped OLEDs. Adv. Funct. Mater. 23, 2329–2337 (2013).

[b14] Liu Y. . Aggregation-induced emissions of fluorenonearylamine derivatives: A new kind of materials for nondoped red organic light-emitting diodes. J. Phys. Chem. C 112, 3975–3981 (2008).

[b15] Qin W. . Construction of efficient deep aggregation-induced emission luminogen from triphenylethene for nondoped organic light-emitting diodes. Chem. Mater. 27, 3892–3901 (2015).

[b16] Sun K. . Novel aggregation-induced emission and thermally activated delayed fluorescence materials based on thianthrene-9,9′,10,10′-tetraoxide derivatives. RSC Adv. 6, 22137–22143 (2016).

[b17] Wang H. . Novel thermally activated delayed fluorescence materials-thioxanthone derivatives and their applications for highly efficient OLEDs. Adv. Mater. 26, 5198–5204 (2014). 24903266 10.1002/adma.201401393

[b18] Xie Z. . White-light emission strategy of a single organic compound with aggregation-induced emission and delayerd fluorescence properties. Angew. Chem. Int. Ed. 54, 7181–7184 (2015). 10.1002/anie.201502180 25925015

[b19] Lee I. H., Song W. & Lee J. Y. Aggregation-induced emission type thermally activated delayed fluorescent materials for high efficiency in non-doped organic light-emitting diodes. Org. Electron. 29, 22–26 (2016).

[b20] Gan S. . Integration of aggregation-induced emission and delayed fluorescence into electronic donor-acceptor conjugates. J. Mater. Chem. C 4, 3705–3708 (2016).

[b21] Yao L. . Highly efficient near-infrared organic light-emitting diode based on a butterfly-shaped donor-acceptor chromophore with strong solid-state fluorescence and a large proportion of radiative excitons. Angew. Chem. 126, 2151–2155 (2014). 10.1002/anie.201308486 24453193

[b22] Tao Y. . Thermally activated delayed fluorescence materials towards the breakthrough of organoelectronics. Adv. Mater. 26, 7931–7958 (2014). 25230116 10.1002/adma.201402532

[b23] Lee S. Y., Yasuda T., Yang Y. S., Zhang Q. & Adachi C. Luminous butterflies: Efficient exciton harvesting by benzophenone derivatives for full-color delalyed fluorescence OLEDs. Angew. Chem. 126, 6520–6524 (2014). 10.1002/anie.201402992 24839234

[b24] Uoyama H., Goushi K., Shizu K., Nomura H. & Adachi C. Highly efficient organic light-emitting diodes from delayed fluorescence. Nature 492, 234–238 (2012). 23235877 10.1038/nature11687

[b25] Lee J. . Oxadiazole- and triazole-based highly-efficient thermally activated delayed fluorescence emitters for organic light-emitting diodes. J. Mater. Chem. C 1, 4599–4604 (2013).

[b26] Hwang S.-H., Moorefield C. N. & Newkome G. R. Dendritic macromolecules for organic light-emitting diodes. Chem. Soc. Rev. 37, 2543–2557 (2008). 18949125 10.1039/b803932c

[b27] Zhang B. . High-efficiency single emissive layer white organic light-emitting diodes based on solution-processed dendritic host and new orange-emitting iridium complex. Adv. Mater. 24, 1873–1877 (2012). 22410940 10.1002/adma.201104758

[b28] Kimoto A., Cho J.-S., Higuchi M. & Yamamoto K. Synthesis and asynmmetrically arranged dendrimers with a carbazole Dendron and a phenylazomethine dendron. Macromolecules 37, 5531–5537 (2004).

[b29] Prachumrak N. . Synthesis and characterization of carbazole dendrimers as solution-processed high *T*_g_ amorphous hole-transporting materials for electroluminescent devices. Eur. J. Org. Chem. 6619–6628 (2013). 10.1039/c2cc16878b 22301677

[b30] Albrecht K., Matsuoka K., Fujita K. & Yamamoto K. Carbazole dendrimers as solution-processable thermally activated delayed-fluorescence materials. Angew. Chem. Int. Ed. 54, 5677–5682 (2015). 10.1002/anie.201500203 25753430

[b31] Hirata S. . Highly efficient blue electroluminescence based on thermally activated delayed fluorescence. Nat. Mater. 14, 330–336 (2015). 25485987 10.1038/nmat4154

[b32] Li Y., Xie G., Gong S., Wu K. & Yang C. Dendronized delayed fluorescence emitters for non-doped, solution-processed organic light-emitting diodes with high efficiency and low efficiency roll-off simultaneously: Two parallel emissive channels. Chem. Sci. 7, 5441–5447 (2016). 10.1039/c6sc00943c PMC6021754 30034683

[b33] Lee S. Y., Yasuda T., Nomura H. & Adachi C. High-efficiency organic light-emitting diodes utilizing thermally activated delayed fluorescence from triazine-based donor-accepter hybrid molecules. Appl. Phys. Lett. 101, 093306 (2012).

[b34] Albrecht K. & Yamamoto K. Dendritic structure having a potential gradient: New synthesis and properties of carbazole dendrimers. J. Am. Chem. Soc. 131, 2244–2251 (2009). 19175324 10.1021/ja807312e

[b35] Yanai T., Tew D. P. & Handy N. C. A new hybrid exchange-correlation functional using the coulomb-attenuating method (CAM-B3LYP). Chem. Phys. Lett. 393, 51–57 (2004).

[b36] Mataga N., Kaifu Y. & Koizumi M. Solvent effects upon fluorescence spectra and the dipolemoments of excited molecules. Bull. Chem. Soc. J. 29, 465–470 (1956).

[b37] Reichardt C. Solvatochromic dyes as solvent polarity indicators. Chem. Rev. 94, 2319–2358 (1994).

[b38] Kapturkiewicz A. & Nowacki J. Properties of the intramolecular excited charge-transfer states of carbazol-9-yl derivatives of aromatic ketones. J. Phys. Chem. A 103, 8145–855 (1999).

[b39] Zhang Q. . Anthraquinone-based intramolecular charge-transfer compounds: Computational molecular design, thermally activated delayed fluorescence, and highly efficient red electroluminescence. J. Am. Chem. Soc. 136, 18070–18081 (2014). 25469624 10.1021/ja510144h

[b40] Luo J. . Aggregation-induced emission of 1-methyl-1,2,3,4,5-pentaphenylsilole. Chem. Commun. 1740–1741 (2001). 10.1039/b105159h 12240292

[b41] Avdeenko A. A., Dobrovolskaya T. L., Kultchitsky V. A., Naboikin Y. V. & Pakulov S. N. Temperature dependence of luminescence decay time of benzyl. J. Lumin. 11, 331–337 (1976).

[b42] Rineke S. . Highly efficient, dual state emission from an organic semiconductor. Appl. Phys. Lett. 103, 093302 (2013).

[b43] Reineke S. & Baldo M. A. Room temperature triplet state spectroscopy of organic semiconductors. Sci. Rep. 4, 3797 (2014). 24445870 10.1038/srep03797 PMC3896913

[b44] Deol H., Pramanik S., Kumar M., Khan I. A. & Bhalla V. Supramolecular ensemble of a TICT-AIEE active pyrazine derivativeand CuO NPs: A potential photocatalytic system for sonogashira couplings. ACS catal. 6, 3771–3783 (2016).

[b45] Mazumdar P., Das D., Sahoo G. P., Salgado-Morán G. & Misra A. Aggregation induced emission enhancement of 4,4′-bis(diethylamino)benzophenone with an exceptionally large blue shift and its potential use as glucose sensor. Phys. Chem. Chem. Phys. 17, 3343–3354 (2015). 25525803 10.1039/c4cp03772c

[b46] Hu R. . Twisted intramolecular charge transfer and aggregation-induced emission of BODIPY derivatives. J. Phys. Chem. C 113, 15845–15853 (2009).

[b47] Zhang J., Xu B., Chen J., Wang L. & Tian W. Oligo(phenothiazine)s: Twisted intramolecular charge transfer and aggregation-induced emission. J. Phys. Chem. C 117, 23117–23125 (2013).

[b48] Jang J. G., Ji H. J., Kim H. S. & Jeong J. C. TPBI:FIrpic organic light emitting devices with the electron transport layer of Bphen/Alq_3_. Curr. Appl. Phys. 11, 5251–5254 (2011).

[b49] Kim J. W. . Study of sequential Dexter energy transfer in high efficient phosphorescent white organic light-emitting diodes with single emissive layer. Sci. Rep. 4, 7009; doi: 10.1038/srep07009 (2014). 25388087 PMC4228348

