# Supplementary material for: Mulifunctional Dendritic Emitter: Aggregation-Induced Emission Enhanced, Thermally Activated Delayed Fluorescent Material for Solution-Processed Multilayered Organic Light-Emitting Diodes
Source: Sci Rep. 2017 Jan 31;7:41780. doi: 10.1038/srep41780 (PMC5282576; doi:10.1038/srep41780)
Supplement: Supporting Information [file srep41780-s1.pdf]

Supporting Information

Mulifunctional Dendritic Emitter: Aggregation-Induced Emission Enhanced, Thermally Activated Delayed Fluorescent Material for Solution-Processed Multilayered Organic Light-Emitting Diodes

Kenichi Matsuoka[1], Ken Albrecht[2], Kimihisa Yamamoto[2*] & Katsuhiko Fujita[1*]

[1]Institute for Materials Chemistry and Engineering, Kyushu University, 6-1 Kasuga koen, Kasuga, Fukuoka 816-8580, Japan

[2]Laboratory for Chemistry and Life Science, Tokyo Institute of Technology, 4259 Nagatsuta Midori-ku, Yokohama 226-8503, Japan

General. UV-vis spectra were recorded using a Shimadzu UV-3150 spectrometer at 20 ºC. In case of solution measurements, a quartz cell having a 1cm optical length was used. The room temperature fluorescence spectra and the fluorescence quantum yields were measured by a Hamamatsu Photonics C9920-02 absolute PL quantum yield measurement system at room temperature (excitation wavelength: 380 nm). The fluorescence and phosphorescence spectra (77K, 100 μs delay and integration time 200 μs) in Supplementary Fig. S5 were measured by a HORIBA JOBIN YVON FluoroMax-4 Spectrometer (excitation wavelength: 365nm), and the sample temperature was controlled with an Optistat DN (Oxford instruments). The PL lifetime measurements were performed using Hamamatsu photonics fluorescence lifetime spectrometer C11367 (Quantaurus-Tau, excitation wavelength of 365nm for 50 ns and 50 μs time range measurement. For 10 ms time range measurement, the excitation wavelength was 370 nm), and the sample temperature was controlled with an Optistat DN (Oxford instruments). HOMO level of the GnB films were measured by photoelectron yield spectroscopy in air using a AC-2 (Riken keiki). Atomic force microscopy (AFM) experiments were carried out by Nanopics 1000 (Seiko instruments).

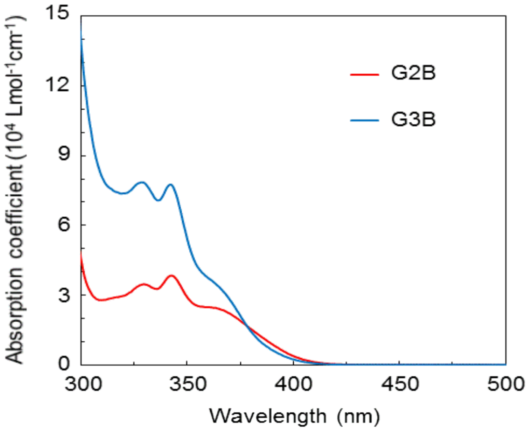

Figure S1. Absorption coefficient of G2B and G3B measured in toluene.

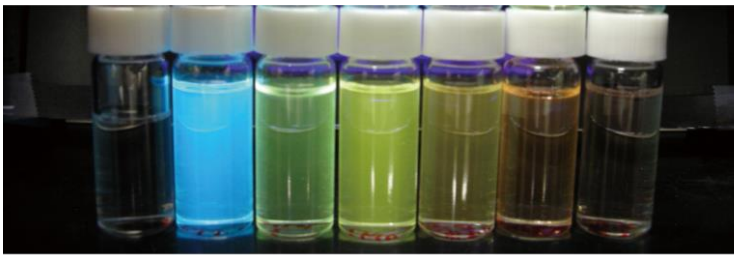

Figure S2. Fluorescent image of G2B in various solvents. Solvents are from the left pentane, toluene, tetrahydrofuran (THF), chloroform, acetone, acetonitrile, and dimethyl sulfoxide (DMSO).

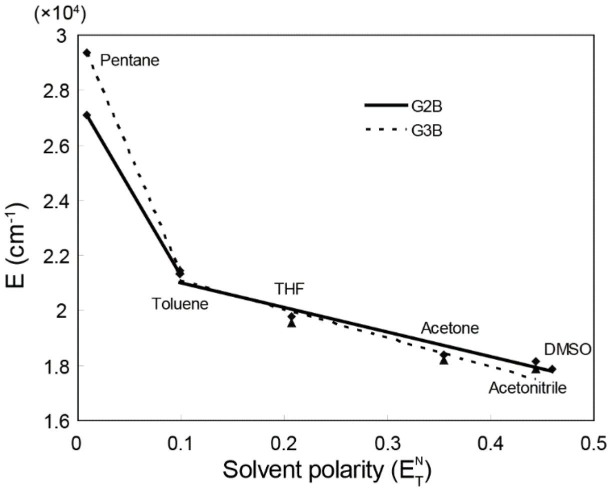

Figure S3. Lippert-Mataga plot of G2B and G3B.

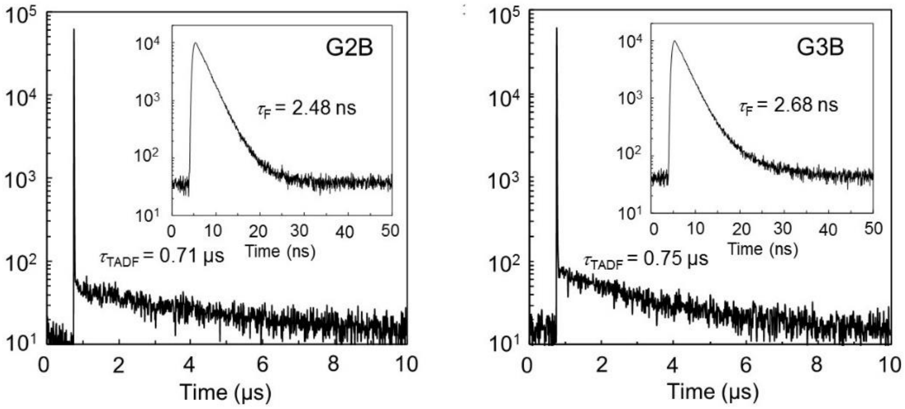

Figure S4. Transient PL decay curves of G2B and G3B in 10 μM toluene solution at room temperature. Prompt and delayed components were fit by single exponential model.

Table S1. Physical parameters of G2B and G3B in toluene solution.

	G2B	G3B
Φ	0.236	0.147
Φ_F	0.231	0.136
Φ_{TADF}	0.005	0.011
τ_F (ns)	2.48	2.68
τ_{TADF} (μs)	0.71	0.75
k_F (s^{-1})	9.31 x 10^7	5.08 x 10^7
k_{IC} (s^{-1})	3.02 x 10^8	2.95 x 10^8
k_{ISC} (s^{-1})	8.54 x 10^6	2.90 x 10^7
Φ_{IC}	0.75	0.79
Φ_{ISC}	0.02	0.08
k_{TADF} (s^{-1})	3.03 x 10^5	1.97 x 10^5
ΔE_{ST} (eV)	0.120	0.115

ΔE_{ST} value and related physical parameters were calculated based on the following list of equations according to reference 39. IC : internal conversion, ISC : intersystem crossing

$k_F = \Phi_F / \tau_F$

$\Phi = k_F/(k_F + k_{IC})$

$\Phi_F = k_F/(k_F + k_{IC} + k_{ISC})$

$\Phi_{IC} = k_{IC}/(k_F + k_{IC} + k_{ISC})$

$\Phi_{ISC} = 1 - \Phi_F - \Phi_{IC} = k_{ISC}/(k_F + k_{IC} + k_{ISC})$

$k_{TADF} = \Phi_{TADF} / \Phi_{ISC} \tau_{TADF}$

$k_{TADF} = (1/3)[k_F \exp(-\Delta E_{ST}/RT)]$

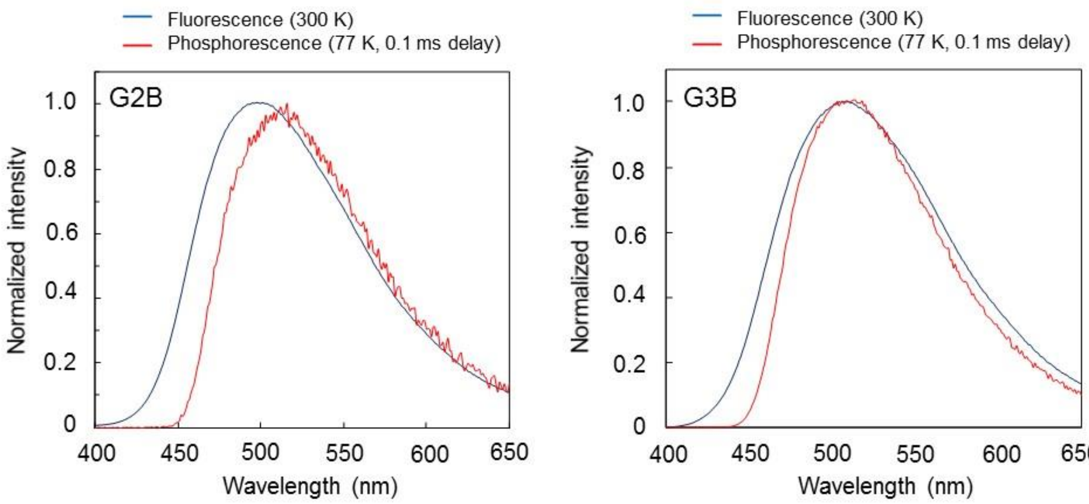

Figure S5. PL and phosphorescence spectra of G2B and G3B neat films.

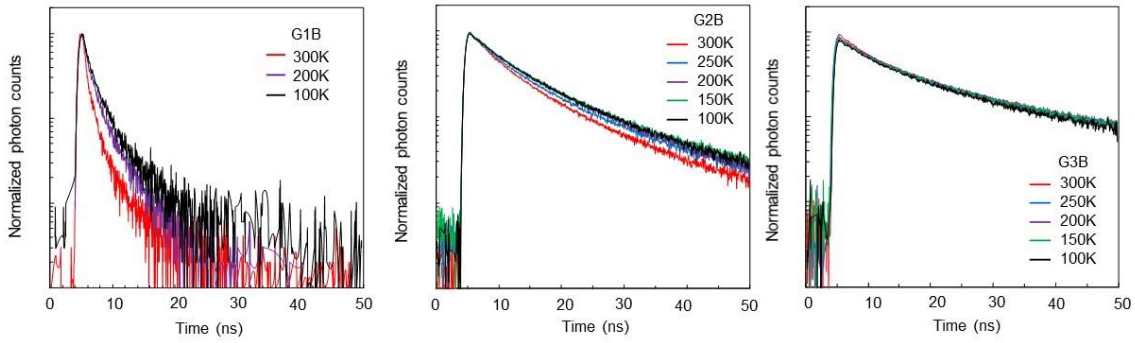

Figure S6. Temperature dependent transient PL decay curves of GnB films in 50 ns time range.

Table S2. PL lifetime and quantum yields of GnB neat films at various temperatures.

Sample	Φ	τ_F (ns)[a]	Φ_F	τ_{TADF} (μs)	Φ_{TADF}
G1B - 100 K	---	1.0	---	---	0
200 K	---	1.8	---	---	0
300 K	0.033	2.1	0.033	2.13	4.0×10^{-5}
G2B - 100 K	0.361	12.3	0.339	0.45	0.022
150 K	0.411	12.2	0.296	10.9	0.115
200 K	0.401	12.0	0.265	9.58	0.136
250 K	0.367	11.2	0.213	6.06	0.154
300 K	0.334	10.4	0.200	2.69	0.134
G3B - 100 K	0.878	21.4	0.279	3.70 [b]	0.599
150 K	0.709	22.4	0.207	3.87 [b]	0.502
200 K	0.456	22.5	0.129	3.54 [b]	0.327
250 K	0.329	22.4	0.104	2.64 [b]	0.225
300 K	0.211	20.8	0.075	3.35 [b]	0.136

[a] Averaged lifetime calculated by $\tau_{av} = \Sigma A_i*(\tau_i)^2/\Sigma A_i*\tau_i$ from the results of bi-exponential fitting in 50 ns-range decay profile (Fig. S6).

[b] Averaged lifetime ($\tau_{av} = \Sigma A_i*(\tau_i)^2/\Sigma A_i*\tau_i$) of relatively long lifeitme components ($\tau > 0.1$ μs) calculated from the results of tri-exponential fitting in 10 μs-range decay profile. (Fig. 3)

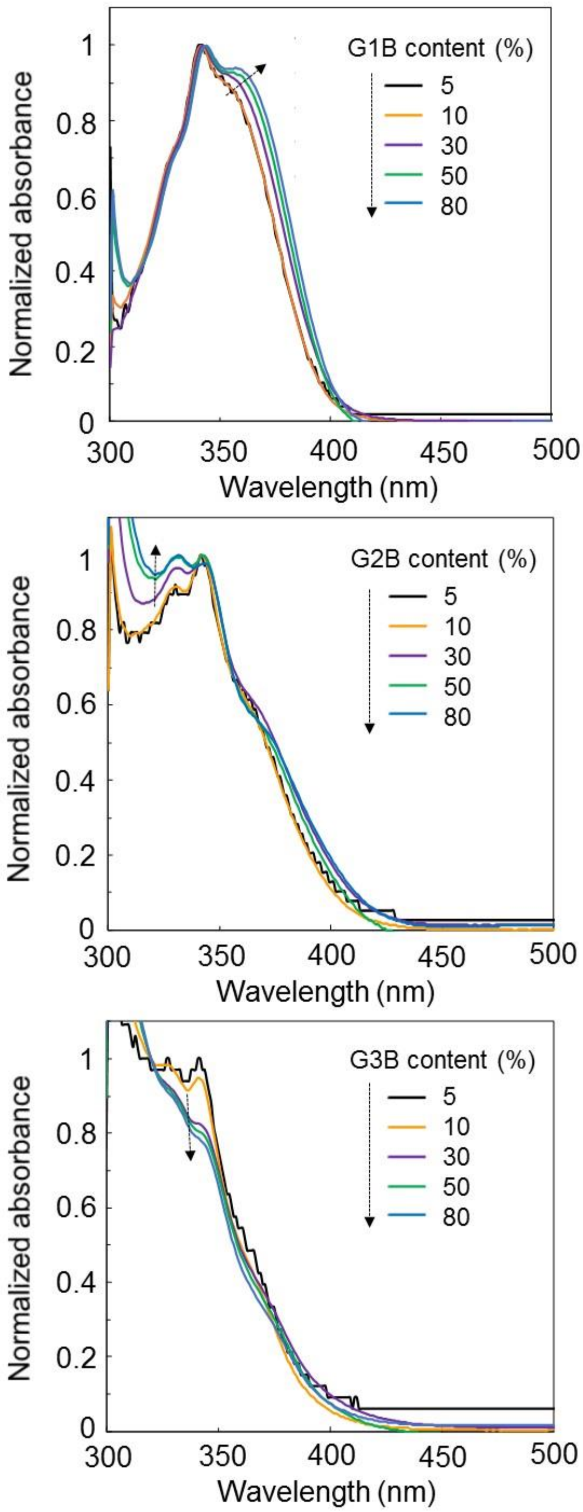

Figure S7. Normalized UV-vis spectra of GnB-PMMA composite films.

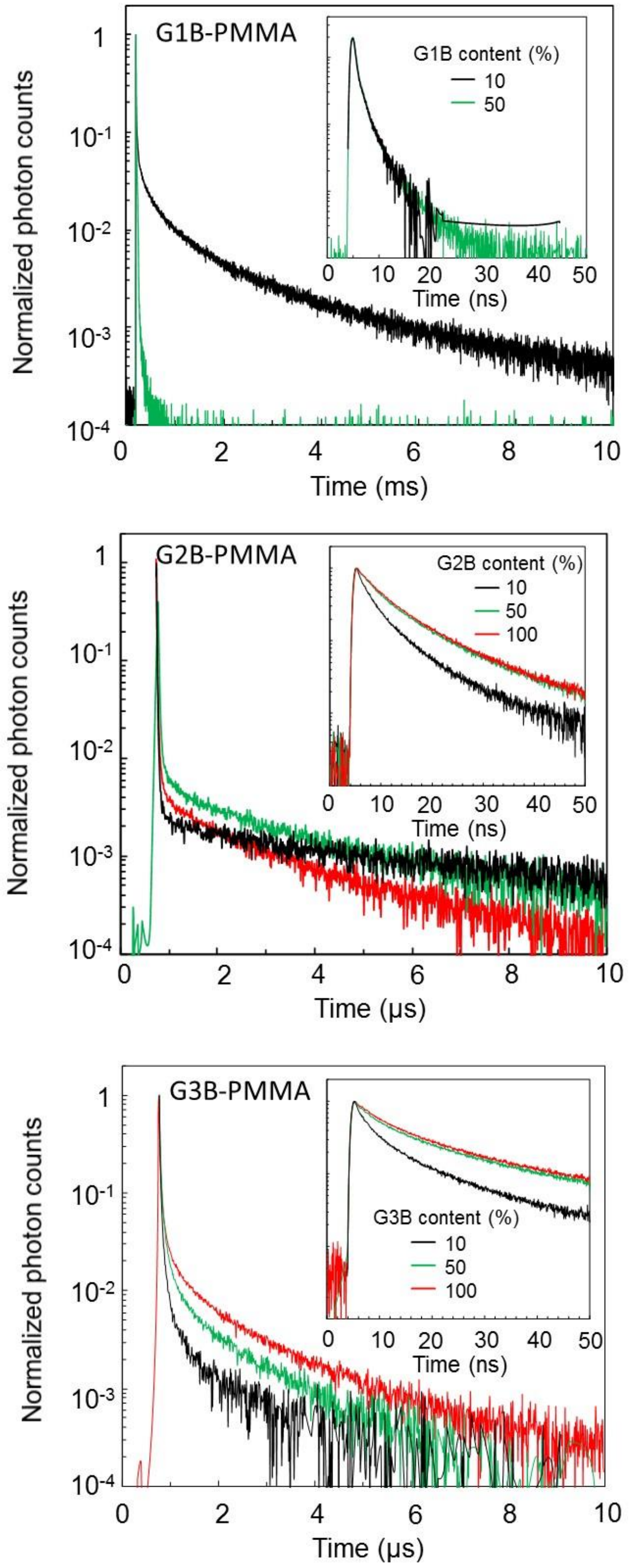

Figure S8. Transient PL decay curves of GnB-PMMA composite films at room temperature.

Table S3. PL lifetimes and quantum yields of GnB-PMMA composite films (300 K).

Sample	Φ	τ_F (ns)[a]	Φ_F	τ_{TADF} (μs)	Φ_{TADF}
G1B neat film	0.033	2.1	0.033	2.13	4.0 x 10^{-5}
50 wt.% in PMMA	0.03	1.8	0.03	--- [b]	~0
10 wt.% in PMMA	0.131	1.2	0.043	1.76 x10^3 [b]	0.088
G2B neat film	0.334	10.4	0.200	2.69	0.134
50 wt.% in PMMA	0.248	10.4	0.150	2.88	0.098
10 wt.% in PMMA	0.205	6.0	0.156	2.97	0.049
G3B neat film	0.211	20.8	0.075	3.35 [c]	0.136
50 wt.% in PMMA	0.157	19.4	0.106	0.534	0.051
10 wt.% in PMMA	0.127	11.8	0.101	0.174	0.026

[a] Averaged lifetime calculated by $\tau_{av} = \Sigma A_i*(\tau_i)^2/\Sigma A_i*\tau_i$ from the results of bi-exponential fitting in 50 ns-range decay profile (inset of Fig. S8).

[b] Lifetime calculated from the 10 ms-range decay profile.

[c] Averaged lifetime ($\tau_{av} = \Sigma A_i*(\tau_i)^2/\Sigma A_i*\tau_i$) of relatively long lifeitme components (τ > 0.1 μs) calculated from the results of tri-exponential fitting in 10 μs-range decay profile.

In case of G1B, very long lifetime (τ = 1.76 ms) component was observed at 10 wt.% G1B content. This value is relatively longer than the value (0.71 ms) measured in DPEPO matrix reported in reference 23.

Table S4. Physical parameters of G2B-, and G3B-PMMA composite films (300 K).

Sample	k_F (10^7 s^{-1})	k_{IC} (10^7 s^{-1})	Φ_{IC}	k_{ISC} (10^7 s^{-1})	Φ_{ISC}	k_{TADF} (10^4 s^{-1})
G2B neat film	1.91	3.82	0.40	3.88	0.40	12.4
50 wt.% in PMMA	1.46	4.37	0.45	3.83	0.40	8.69
10 wt.% in PMMA	2.61	10.1	0.61	3.99	0.24	6.89
G3B neat film	0.36	1.34	0.28	3.10	0.65	6.30
50 wt.% in PMMA	0.54	2.92	0.57	1.68	0.33	29.4
10 wt.% in PMMA	0.86	5.88	0.70	1.72	0.20	72.9

*Each parameter was calculated by the same method used in Table S1.

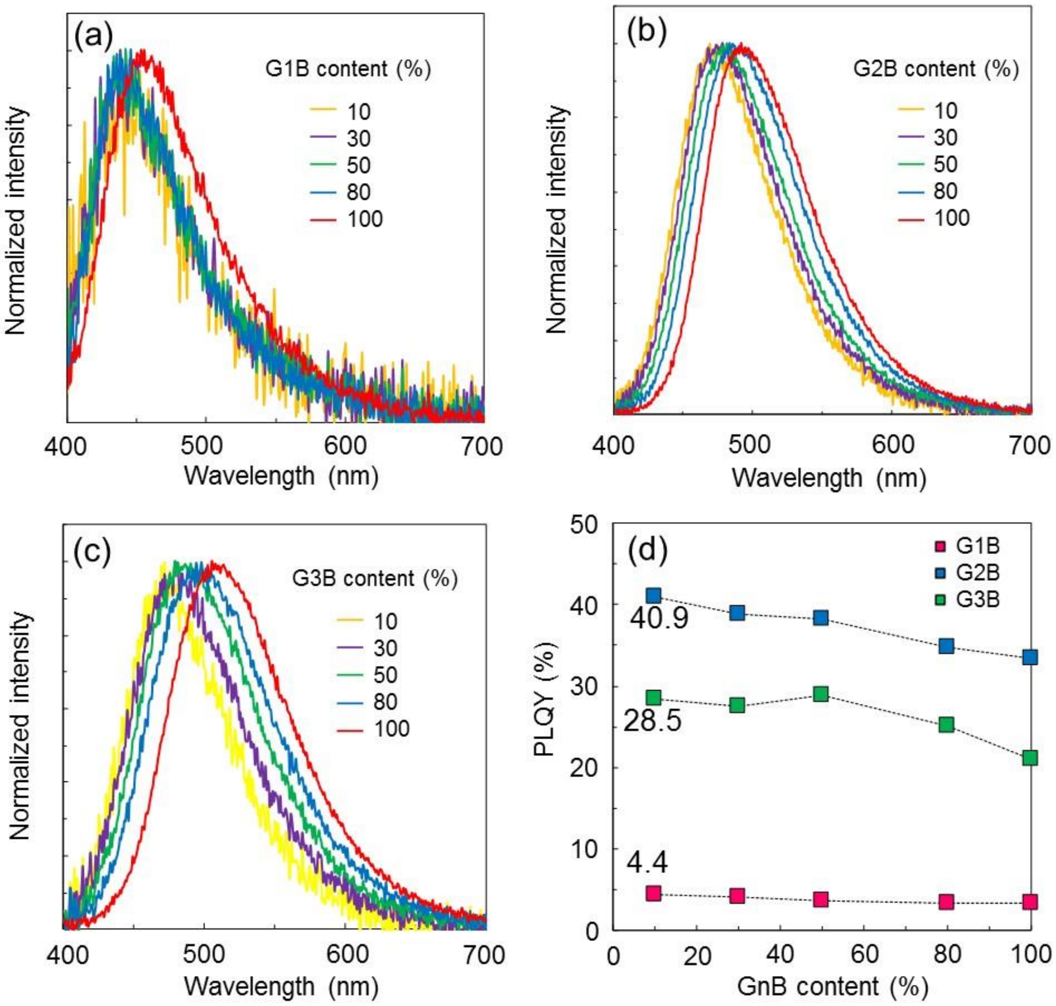

Figure S9. Normalized PL spectra of (a) G1B-mCP, (b) G2B-mCP, (c) G3B-mCP composite films (excited at 380 nm). (d) PLQY of GnB-mCP composite films vs. GnB content.

The composite films of mCP and GnB were prepared from the corresponding toluene solutions by spincoating on glass substrates. mCP and G1B have very similar triplet energy of 2.90 eV and 2.89 eV, respectively. PLQY of G1B in mCP matrix was, therefore, kept low likely due to the quenching of TADF emission by Dexter energy transfer to mCP.

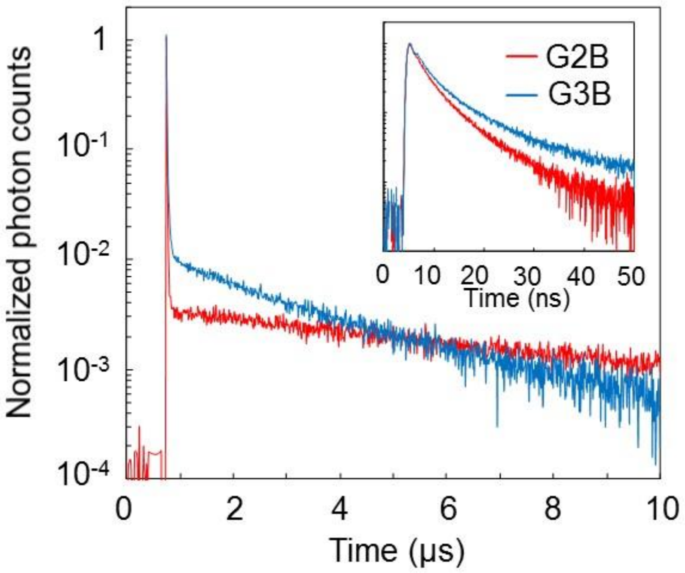

Figure S10. Transient PL decay curves of 10 wt.% GnB doped mCP films at room temperature.

Table S5. Physical parameters of 10 wt.% G2B and G3B doped in mCP matrix (300 K).

	G2B (10 wt.% in mCP)	G3B (10 wt.% in mCP)
Φ	0.409	0.285
Φ_F	0.154	0.095
Φ_{TADF}	0.255	0.190
τ_F (ns)[a]	5.53	4.72
τ_{TADF} (μs)	4.73	1.90
k_F (s^{-1})	2.78 x 10^7	2.01 x 10^7
k_{IC} (s^{-1})	4.02 x 10^7	5.05 x 10^7
k_{ISC} (s^{-1})	1.13 x 10^8	1.41 x 10^8
Φ_{IC}	0.22	0.24
Φ_{ISC}	0.62	0.67
k_{TADF} (s^{-1})	8.64 x 10^4	1.50 x 10^5
ΔE_{ST} (eV)	0.12	0.10

*Each parameter was calculated by the same method used in Table S1.

[a] Averaged lifetime calculated by $\tau_{av} = \Sigma A_i*(\tau_i)^2/\Sigma A_i*\tau_i$ from the results of bi-exponential fitting in 50 ns-range decay profile (inset of Fig. S10).

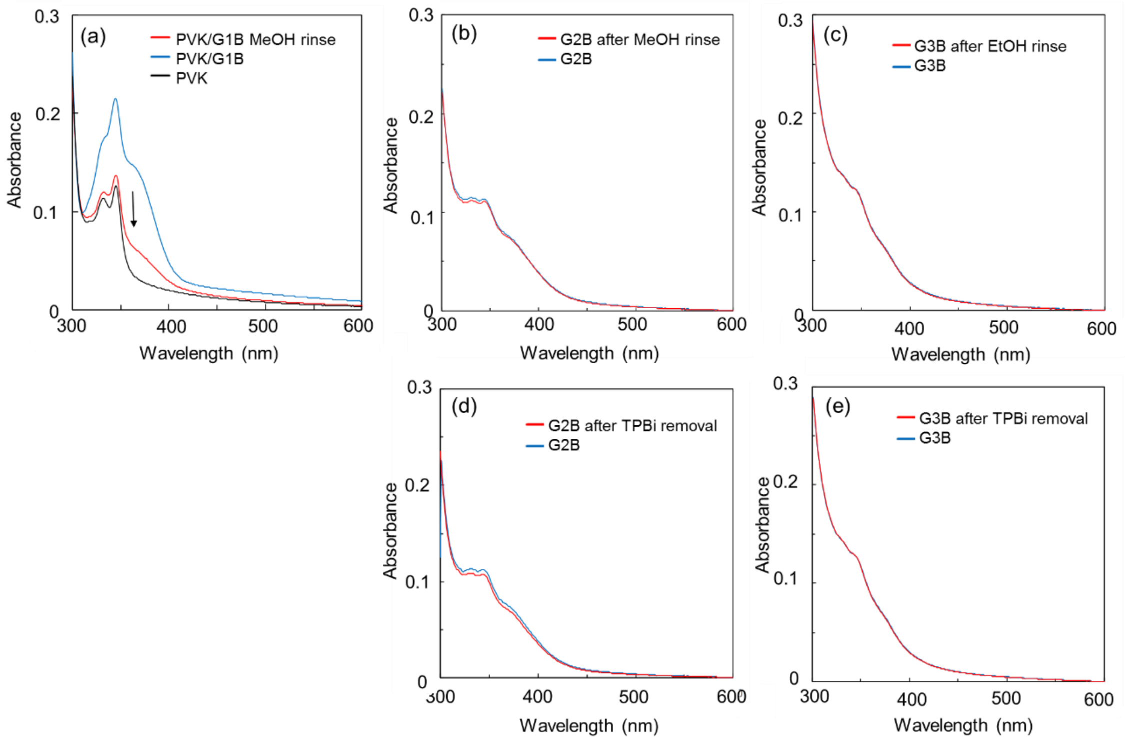

Figure S11. (a-c) UV-vis spectra of GnB films before and after alcohol rinsing. (d,e) UV-vis spectra of GnB films before TPBi layer deposition by spincoating and after TPBi layer deposition followed by the removal of TPBi layer by alcohol rinsing.

GnB films were spincoated from toluene solutions (7 mg/ml, ~30 nm) on PVK (ca. 20 nm)/glass or glass substrates. The GnB films were then spinrinsed by methanol (MeOH) or ethanol (EtOH) (Fig. S11a-c). PVK layer was necessary for G1B to form a uniform thin film. In case of Figure S11d and e, TPBi layer was first spincoated on GnB films in the same manner as OLED fabrication, then the TPBi layers were washed away by the alcohols used for the TPBi deposition.

As can be seen in Fig. S11a, G1B layer was severely dissolved by alcohol treatment. In contrast, G2B and G3B films seemed to be almost intact after the alcohol spinrinsing or TPBi layer deposition/removal procedure. In case of G2B film, however, its absorbance after alcohol rinsing or TPBi removal procedure reproducibly appeared slightly lower than that of as-prepared film. The absorbance difference was up to ~ 0.002 for MeOH rinsing and ~ 0.008 for TPBi removal procedure at 350 nm, where absorbance of the as-prepared film was ca. 0.1, implying that a small part of G2B layer could be washed away by alcohol (MeOH) rinsing and during the TPBi layer deposition by spincoating in OLED fabrication.

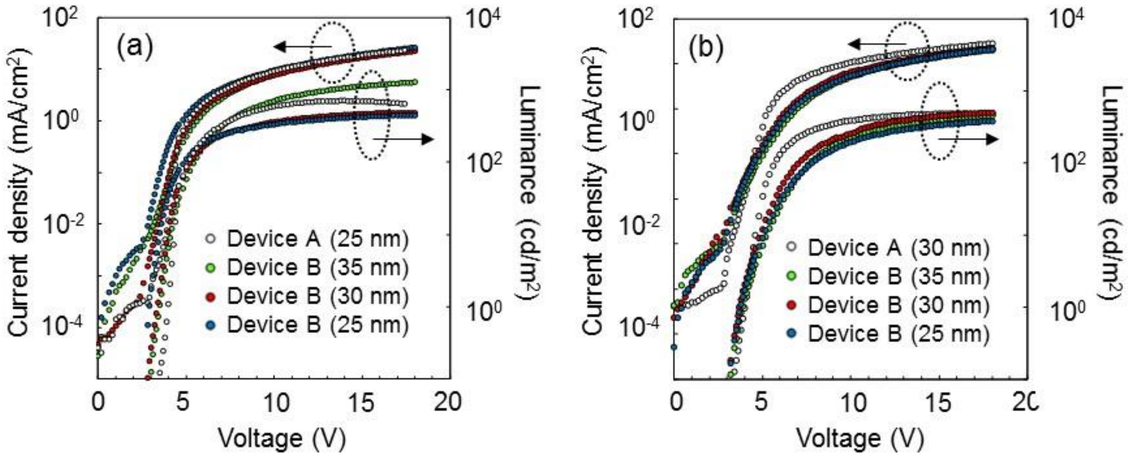

Figure S12. Current density-voltage-luminance curves of OLED devices (a) G2B and (b) G3B.

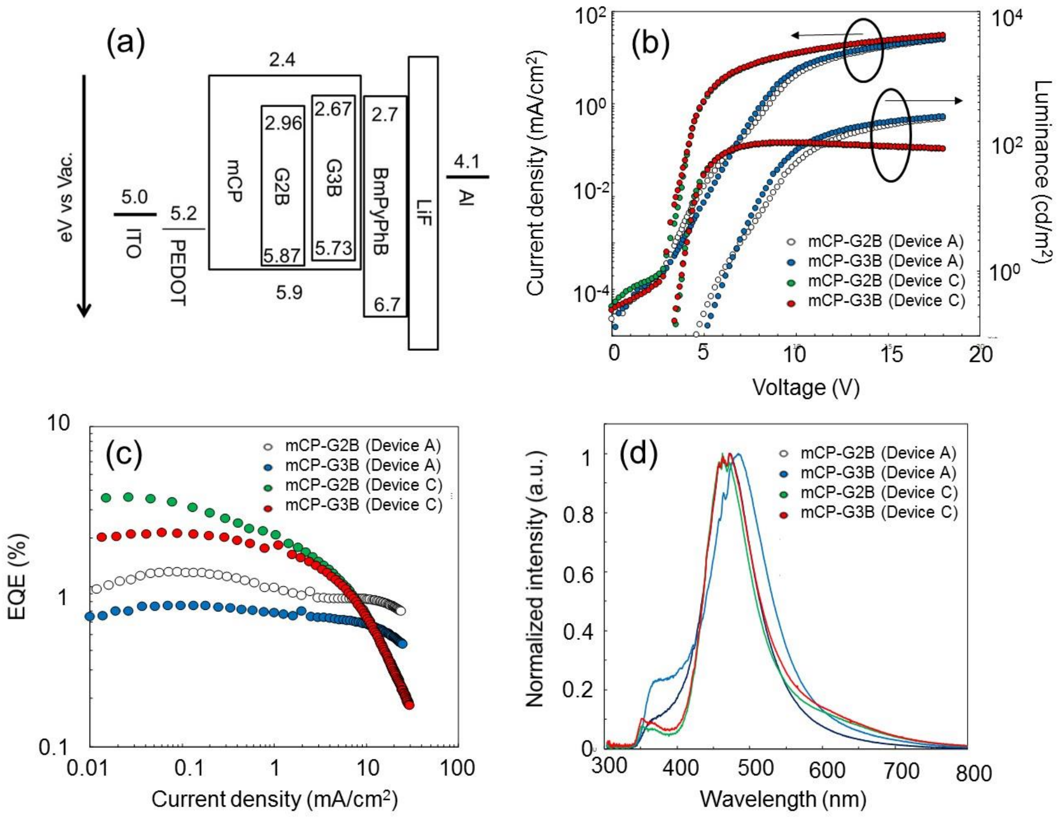

Figure S13. (a) Energy diagram of OLED device C. (b) current density-voltage-luminance curves, (c) EQE-current density characteristics, (d) EL spectra measured at 10 mA/cm^2 of OLEDs with mCP-GnB EMLs.
